# Microparticles vs. Macroparticles as Curcumin Delivery Vehicles: Structural Studies and Cytotoxic Effect in Human Adenocarcinoma Cell Line (LoVo)

**DOI:** 10.3390/molecules26196056

**Published:** 2021-10-06

**Authors:** Joanna Wezgowiec, Marta Tsirigotis-Maniecka, Jolanta Saczko, Mieszko Wieckiewicz, Kazimiera A. Wilk

**Affiliations:** 1 Department of Experimental Dentistry, Wroclaw Medical University, 50-425 Wroclaw, Poland; joanna.wezgowiec@umw.edu.pl; 2 Department of Engineering and Technology of Chemical Processes, Wroclaw University of Science and Technology, Wybrzeze Wyspianskiego 27, 50-370 Wroclaw, Poland; kazimiera.wilk@pwr.edu.pl; 3 Department of Molecular and Cellular Biology, Wroclaw Medical University, 50-556 Wroclaw, Poland; jolanta.saczko@umw.edu.pl

**Keywords:** curcumin, turmeric, sodium alginate, chitosan, gelatin, drug delivery system, natural product, phytotherapeutic, colon cancer, colorectal cancer

## Abstract

This study aimed to characterize the hydrogel micro- and macro-particles designed to deliver curcumin to human colon cancer cells (LoVo). Six series of vehicles based on sodium alginate (micro- and macro-particles, uncoated, coated with chitosan or gelatin) were synthesized. The uncoated microparticles were fabricated using an emulsion-based technique and the uncoated macroparticles with an extrusion technique, with both coupled with ionotropic gelation. The surface morphology of the particles was examined with scanning electron microscopy and the average size was measured. The encapsulation efficiency, moisture content, and swelling index were calculated. The release of curcumin from the particles was studied in an experiment simulating the conditions of the stomach, intestine, and colon. To evaluate the anticancer properties of such targeted drug delivery systems, the cytotoxicity of both curcumin-loaded and unloaded carriers to human colon cancer cells was assessed. The microparticles encapsulated much less of the payload than the macroparticles and released their content in a more prolonged manner. The unloaded carriers were not cytotoxic to LoVo cells, while the curcumin-loaded vehicles impaired their viability—more significantly after incubation with microparticles compared to macroparticles. Gelatin-coated or uncoated microparticles were the most promising carriers but their potential anticancer activity requires further thorough investigation.

## 1. Introduction

Nature remains an abundant source of biologically active compounds that are possible to apply for various purposes in many medical specialties, especially for the treatment of cancer and infectious diseases [1]. However, to fully benefit from their advantageous properties, challenges such as molecular complexity, low solubility, and instability must be overcome [2].

Among the multitude of natural substances, curcumin attracts a lot of attention due to its versatile bioactivity. This active ingredient of turmeric—an Indian spice derived from the roots of the *Curcuma longa* plant—is known for its anti-inflammatory, antioxidant, and anticancer properties [3,4]. In terms of application for anticancer treatment, it was demonstrated that curcumin was able to suppress the proliferation of several different types of cancer cell lines, including prostate, breast, colorectal, pancreatic and head and neck cancers [5,6]. Its further potential applications, including photodynamic therapy for cancer, are currently being investigated [7].

One of the most promising yet demanding applications of curcumin is its use in colon cancer chemoprevention and therapy [8]. There is evidence suggesting the potential of such an approach due to the ability of curcumin treatment to induce apoptosis and inhibit tumor growth. Ramasamy et al. emphasized the potential of curcumin in lowering tumor recurrence by targeting the colorectal cancer stem cells population and sensitizing them towards anticancer agents [9]. Notwithstanding, the results of clinical studies are not fully satisfactory and there are still some challenges to overcome (e.g., low bioavailability and aqueous solubility) [5]. While several innovative drug delivery systems have been proposed to prepare nanoformulations with curcumin (e.g., Lipocurc™, Meriva^®^, ExoCUR, BCM-95^®^ CG) for this purpose, their safety and effectiveness still require deeper evaluation [10,11,12,13].

The most convenient way to introduce the drug into the body is the oral route as it is the least invasive. Nevertheless, the low water solubility is a major drawback that hinders the oral therapeutic use of curcumin. Due to the low intrinsic dissolution rate, its bioavailability is reduced, and its clinical effectiveness is limited [14]. Curcumin is unstable in the gastrointestinal tract, especially under alkaline conditions; it undergoes unfavorable processing, i.e., decomposition, poor absorption, and rapid metabolism followed by excretion from the body [15]. The specific environment of the gastrointestinal tract requires a special approach to obtain a targeted and tailored system for the delivery of drugs to the colon [16]. One approach aiming to achieve this goal is using encapsulation technology, which enables the entrapment of an unstable substance and its release in a controlled manner [17]. The incorporation of curcumin into tailored delivery systems may provide it with stability and improved solubility, and even protect it during storage [4]. Additionally, such a system may provide controlled delivery and sustained release, achieving a higher concentration of the active compound at the target site, increasing curcumin targeting efficacy.

Biopolymers have multiple advantages that make them particularly useful for encapsulating therapeutic substances—they are derived from renewable sources, economically convenient, and readily available. They are typically biocompatible, biodegradable, and nontoxic as well. Moreover, due to specific functional groups, mainly hydroxyl (i.e., alginate, carrageenan, carboxymethylcellulose) and amines (i.e., chitosan, gelatin), they are highly reactive, which allows them to be employed in various encapsulation systems. It is highly recommended to construct a drug delivery system (DDS) combining safe and inexpensive substances from various groups such as polysaccharides (chitosan) and proteins (gelatin). Such approach provides beneficial features of carriers, including resistance to adverse conditions and the ability to release the payload in a controlled manner. Particularly, chitosan (CHIT) and gelatin (GEL) are of considerable interests among various substances valuable as a building blocks for DDS [18,19]. Being appropriately charged polyelectrolytes capable of electrostatic interactions with the alginate (ALG) core, CHIT and GEL are often selected as coating materials for ALG-based particles and their suitability was confirmed in our previous study [20]. Encapsulation in biopolymer-based vehicles often occurs under mild aqueous conditions, without toxic solvents. The same compound may be encapsulated in a similar delivery system through a different methodological approach [21]. Alginate hydrogels fortified with other biopolymers may find practical application as colon-targeted devices. Sodium alginate/carboxymethyl chitosan–ZnO hydrogel beads have been proposed by Niu et al. as an effective and safe system to deliver diclofenac sodium, a nonsteroidal, anti-inflammatory drug, which is sensitive to gastrointestinal environmental effects and could also cause stomach irritation [22]. Braim et al. designed colon-targeted, pH-triggered lactoferrin-loaded microparticles, effective in protection against *C. difficile* toxin-mediated human intestinal epithelial damage [23]. Phytophamaceutics, as unstable substances, could especially benefit from using encapsulation technology for their smart delivery to the target sites.

The purpose of this study was to design and characterize the hydrogel micro- and macro-particles fabricated to deliver curcumin (CUR) to colon cancer cells. The physicochemical properties of the CUR-loaded particles were examined. The release of CUR from the particles was studied in an experiment simulating the conditions of the stomach (SGF), intestine (SIF), and colon (SCF). Finally, the cytotoxicity of such targeted drug delivery systems to human colon adenocarcinoma cells was assessed.

## 2. Results and Discussion

Six series of micron-sized hydrogel particles based on sodium alginate (ALG), chitosan (CHIT), and gelatin (GEL) were synthesized. To clearly distinguish the particles obtained using an emulsification-based technique from those obtained using an extrusion-based technique, both combined with ionotropic gelation, separate terms have been introduced. Due to the fact that the particles originated from uncoated ones fabricated via the emulsification method were clearly smaller, they were designated as microparticles, while the ones originated from uncoated particles produced by the extrusion method were marked as macroparticles. CHIT or GEL coating was applied onto the uncoated particles using an electrostatic assembly (Table 1). Due to the well-known therapeutic potential of curcumin (CUR), we used it as a model payload and encapsulated it in hydrogel particles [24]. The physicochemical properties of the CUR-loaded particles were examined to determine how the structure of the particle (concentration of the core polymer, presence and type of the external shell) affects its performance in simulated gastrointestinal conditions and its applicability as a carrier that could enhance the bioavailability of curcumin in the treatment of colon cancer.

### 2.1. Characteristics of Micro- and Macro-Particles

CUR-loaded micro- and macro-particles displayed a yellow color corresponding to the natural color of curcumin. The color was uniform throughout the particle structure, suggesting that the payload was dispersed homogenously. The conclusion on the successful encapsulation of CUR was in accordance with the results of Fourier transform infrared spectroscopy studies.

#### 2.1.1. FTIR Studies Results

**Figure 1:**
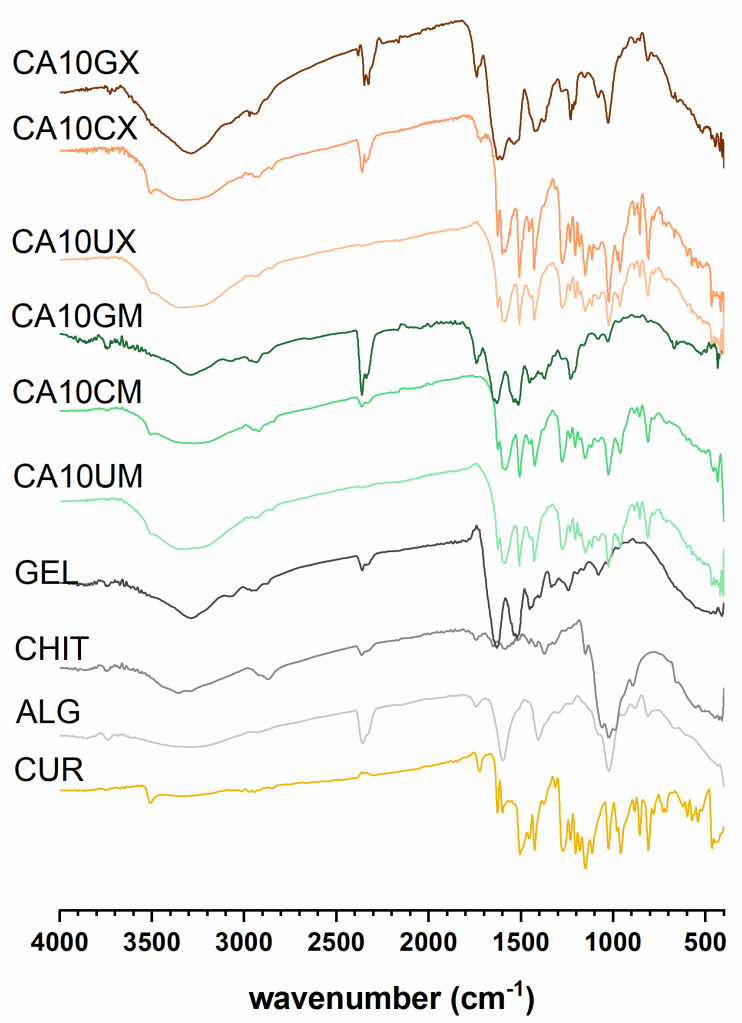
Fourier transform infrared spectroscopy (FTIR) results for curcumin (CUR), sodium alginate (ALG), chitosan (CHIT), gelatin (GEL), and CUR-loaded micro- (CA10UM, CA10CM, CA10GM) and macroparticles (CA10UX, CA10CX, CA10GX) (see Table 1 for details of the particle composition).

The spectra recorded for CUR-loaded particles (Figure 1) revealed multiple bands corresponding to the vibrations of bonds in functional groups present in the CUR structure: a weak signal at 3507–3505 cm^−1^ attributed to phenolic O–H; a sharp, medium peak at 1627–1625 cm^−1^ from stretching vibrations of C=C and C=O in both aromatic and aliphatic structures; a sharp, medium peak at 1512–1503 cm^−1^ ascribed to C=O and aromatic C=C; a sharp, a medium signal at 1235–1230 cm^−1^ from CH; medium peaks at 963–959 cm^−1^ (except CA10GM and CA10GX) and 813–819 cm^−1^ ascribed to vibrations of the C–O bond [25]. Additionally, CHIT or GEL represent characteristic adsorption bands confirming the presence of an outer shell coating the ALG core. Bands around 1530 cm^−1^, identified in the spectra of CHIT and GEL coated particles, were involved in N-H vibrations in the chitosan (amino group) and gelatin (amide group) structures [26]. The intermolecular interactions of GEL and ALG were evidenced by the rearrangement of CA10GM and CA10GX spectra in comparison with the particles of ALG alone—the band in the 3500–3000 cm^−1^ range was narrowed, and the amide I band was formed around 3300 cm^−1^. Signals in the 1458–1453 cm^−1^ range in the spectra of CHIT-coated particles can be attributed to C–N bonds [27]. The discrete signals at 1320 cm^−1^ in the CA10CX spectrum and 1590 cm^−1^ in the CA10CM spectrum can be attributed to C–N stretching in the amide III and N-H bending of the primary amine in CHIT, respectively. An absorption band of about 1150 cm^−1^ corresponds to the asymmetric stretching of the C-O-C bridge in chitosan. The bands at 1056 and 1020 cm^−1^, visible in the spectra of CHIT-coated particles, are from the stretching of the C–O bond [28]. During the encapsulation process, CUR remained stable (Figure S1).

#### 2.1.2. The Encapsulation Efficiency, the Average Size of the Particles and the Moisture Content

**Figure 2:**
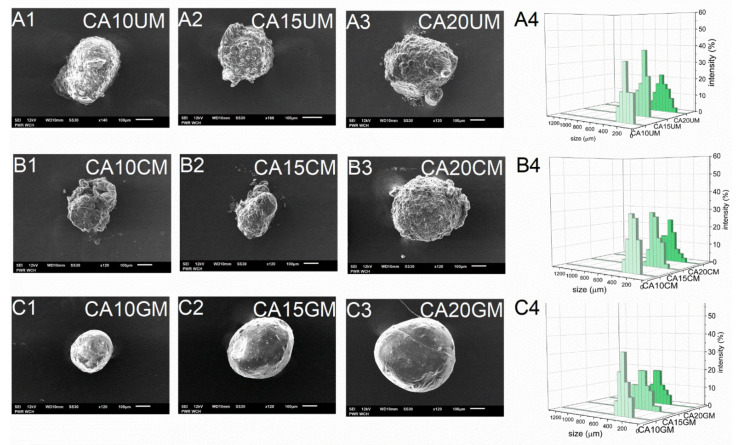
Scanning electron microscopy (**A1**–**A3**,**B1**–**B3**,**C1**–**C3**) and size distribution (**A4**,**B4**,**C4**) images of CUR-loaded microparticles (see Table 1 for details of the particle composition).

**Figure 3:**
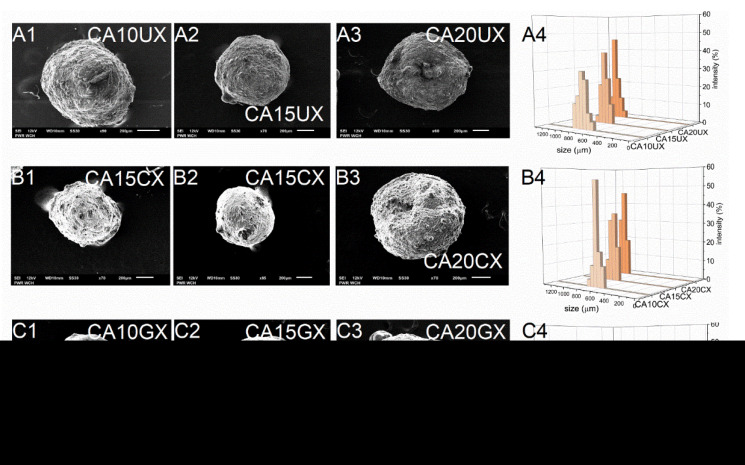
Scanning electron microscopy (**A1**–**A3**,**B1**–**B3**,**C1**–**C3**) and size distribution (**A4**,**B4**,**C4**) images of CUR-loaded macroparticles (see Table 1 for details of the particle composition).

As expected, the encapsulation efficiency (EE) differed significantly between micro- and macro-particles. Regardless of the structural composition, the macroparticles of all types encapsulated over 90% of the payload, however, the microparticles of all types encapsulated only about 60% of it. These differences arose during the fabrication process—encapsulation of CUR in microparticles requires more of the unit processes during which the payload may be lost. Although they carry a relatively smaller amount of payload, in some cases microparticles, when considered drug delivery systems, may be preferred over microparticles. They could make the oral administration of a very potent bioactive substance such as curcumin more precise, even in very small doses, while maintaining control of its release during treatment. The size (D) of the microparticles was 259–429 μm and the size of the macroparticles was 682–1067 μm, and dependent directly on the particle synthesis method. Within each series of particles, D increased along with the concentration of the core polymer, regardless of the shell. Increasing the concentration of ALG led to a less compact particle structure. Moreover, among particles with the same core polymer concentration, the CHIT-coated ones were of the lowest size while the GEL-coated ones were of the highest size (Table 1). The microparticles (Figure 2) showed a slightly less uniform size distribution than the macroparticles (Figure 3), which may be related to the tendency of the hydrogel droplets to aggregate or break during formation.

**Table 1:** Composition and characteristics of the CUR-loaded particles. The results are presented as the means ± S.D.

Particle Type	Particle	C_ALG_^a^ (%)	Coating	EE ^b^ (%)	D ^c^ (μm)	MC ^d^ (%)
microparticles	CA10UM	1.0	**uncoated**	57.1 ± 1.3	259 ± 24	5.7 ± 0.2
	CA15UM	1.5		68.7 ± 0.6	297 ± 48	5.6 ± 0.2
	CA20UM	2.0		63.4 ± 0.7	323 ± 43	7.5 ± 0.2
	CA10CM	1.0	**CHIT ^e^**	60.7 ± 2.1	263 ± 31	6.6 ± 0.2
	CA15CM	1.5		52.2 ± 0.5	278 ± 34	2.0 ± 0.1
	CA20CM	2.0		59.5 ± 5.0	379 ± 50	3.0 ± 0.1
	CA10GM	1.0	**GEL ^f^**	61.8 ± 5.9	317 ± 35	4.2 ± 0.2
	CA15GM	1.5		69.7 ± 2.0	397 ± 43	5.2 ± 0.3
	CA20GM	2.0		58.8 ± 6.2	429 ± 42	5.1 ± 0.3
macroparticles	CA10UX	1.0	**uncoated**	92.6 ± 0.1	772 ± 64	5.6 ± 0.1
	CA15UX	1.5		94.5 ± 0.1	814 ± 40	5.9 ± 0.3
	CA20UX	2.0		93.8 ± 0.4	1009 ± 55	6.2 ± 0.2
	CA10CX	1.0	**CHIT ^e^**	92.4 ± 0.1	682 ± 70	5.2 ± 0.1
	CA15CX	1.5		93.9 ± 0.1	798 ± 46	5.6 ± 0.1
	CA20CX	2.0		96.0 ± 0.1	985 ± 39	5.7 ± 0.2
	CA10GX	1.0	**GEL ^f^**	94.5 ± 0.1	803 ± 70	9.0 ± 0.4
	CA15GX	1.5		97.0 ± 0.1	990 ± 80	7.3 ± 0.3
	CA20GX	2.0		95.7 ± 0.1	1067 ± 63	7.2 ± 0.5

^a^ Sodium alginate concentration [%]; ^b^ Encapsulation efficiency ± S.D.; ^c^ Average size ± S.D.; ^d^ Moisture content ± S.D.; ^e^ Chitosan; ^f^ Gelatin.

#### 2.1.3. SEM Analysis of the Particles Surface Morphology

SEM studies of the particles showed their similar shapes, but their surfaces had a significantly different morphology. Generally, all types of macroparticles and the GEL-coated microparticles were spherical. CA20CM and uncoated microparticles were slightly oval (Figure 2 and Figure 3). Moreover, CA10CM and CA15CM seemed to be shapeless due to an intensive shrinking of the matrix. This effect may be directly related to the number of crosslinks present in the matrix—the more ALG is in the matrix, the more calcium bonds are formed, leading to higher geometrical stability and ultimately a less collapsed structure. The surface of the uncoated and CHIT-coated particles was rough and clumped. The surface of the GEL-coated microparticles was smooth, while that of the GEL-coated macroparticles was more corrugated but still less wrinkled compared to other macroparticles. The particle surface morphology can be strongly influenced by the outer shell composition. Additionally, an increase in the concentration of ALG increases the macroparticle surface roughness. The moisture content (MC) for all particle types was below 10%. Microparticles largely exhibited similar MC values, regardless of the particles structure. For macroparticles, the differences in MC were slightly more pronounced—the lowest MC occurred in CHIT-coated ones and the highest in GEL-coated ones, whereas the concentration of ALG did not exert any effect. GEL-coated macroparticles showed a higher MC as the coating is more viscous than the chitosan coating and retains more water [20].

#### 2.1.4. Swelling Response of the Particles

**Figure 4:**
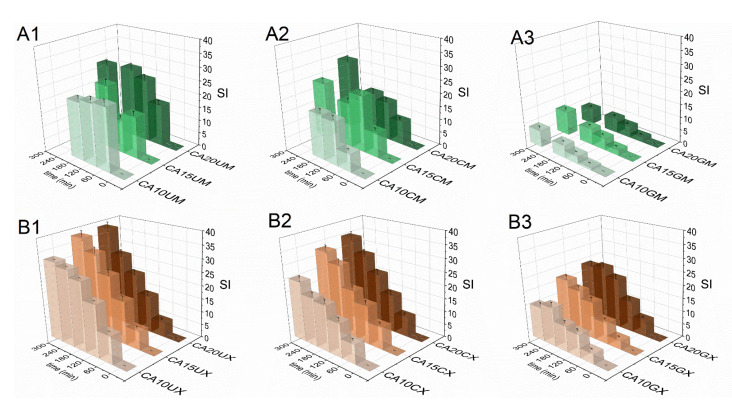
Comparison of the swelling indices (SI) of the CUR-loaded micro- (**A1**–**A3**) and macroparticles (**B1**–**B3**) in Ham’s F-12 medium (see Table 1 for details of the particle composition). The results are presented as the means ± S.D.

The swelling response of the fabricated particles was studied in Ham’s F-12 medium (pH = 7.3) to recognize their behavior in LoVo cell line conditions. Figure 4 shows a comparison of particle swelling along with time. The equilibrium swelling was noted only for microparticles (after 60 min) and they were characterized by lower SI values, however, CA10UM and CA15UM burst after 180 min of the experiment. The macroparticles swelled to a much greater extent, but no rapid disruption was observed. The highest SI values were attained for uncoated particles (SI_CA20UM_ = 27, SI_CA10UX_ = 37), and the lowest for GEL-coated particles (SI_CA20GM_ = 9, SI_CA10GX_ = 20), regardless of size. The applied coating limited the swelling of the core, possibly by reducing the porosity on the particles’ surfaces [29] and/or partially neutralizing the repulsive interactions between alginate chains via oppositely charged functional groups. Higher SIs were noted because of more pronounced repulsive interactions between the ionized carboxylate residues in the alginate backbone along with increasing polymer concentration in the core, due to a denser structure. Additionally, particles with a lower polymer content in the core have a more compact structure, which makes it more difficult for the medium to diffuse into the matrix, which limits swelling. Thus, a similar relationship exists between the concentration of the core polymer and SI, i.e., SI values increase along with the amount of ALG. Nevertheless, a more stable structure could be beneficial for extending the contact time between the particles and the gastrointestinal environment.

### 2.2. Curcumin Release Profiles and Release Kinetic Studies

**Figure 5:**
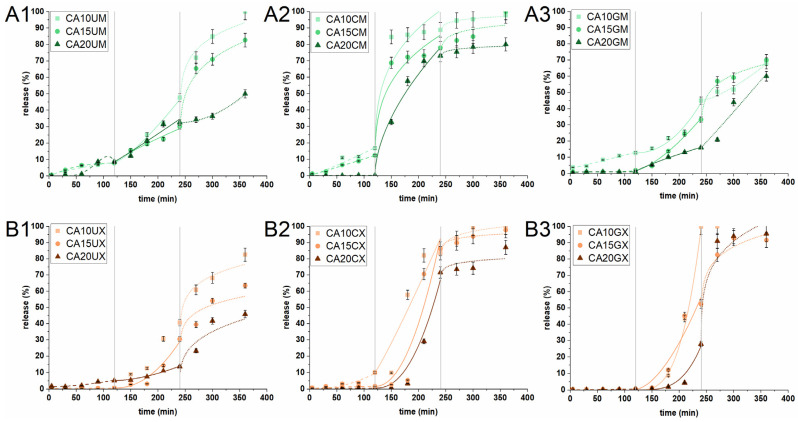
*In vitro* cumulative release profiles of CUR from (**A1**) uncoated microparticles, (**A2**) CHIT-coated microparticles, (**A3**) GEL-coated microparticles, (**B1**) uncoated macroparticles, (**B2**) CHIT-coated macroparticles, and (**B3**) GEL-coated macroparticles under conditions simulating the stomach (0–120 min) (SGF), intestines (121–240 min) (SIF) and colon (241–360 min) (SCF). Lines correspond to CUR release profiles that best fit the selected kinetic models: dashed in SGF, solid in SIF, dotted for SCF (see Table 1 for details of the particle composition). The results are presented as the means ± S.D.

The release of CUR from the particles was studied in an experiment simulating the conditions (composition, temperature, pH, and residence time) of the stomach (SGF), intestine (SIF) and colon (SCF). Three different sequential experimental conditions determine whether the prepared carriers can deliver curcumin to colon cancer sites. Figure 5 shows the *in vitro* release profiles. The amount of CUR released in SGF from macromolecules of all types and microparticles with the highest ALG content was found to be negligible (<2%). This behavior is highly desirable as the chemically unstable payload can degrade under the harsh conditions of the stomach. The remaining microparticles released up to 16% of their charge; the molecules may have clustered close to the surface of the particles. This suggests that most of the encapsulated CUR would either be released in the intestines (for absorption) or transferred for further release in the colon (i.e., to support colon cancer therapy). In the next stage of the experiment (in SIF), a much higher amount of CUR was released—up to 100% for CA10GX. The release was initiated due to the change in pH and ionic strength of the acceptor medium. The increase in pH above the pKa of ALG initiated the deprotonation of carboxyl groups and introduced repulsive interactions. This phenomenon led to swelling and loosening of the particle structure, promoting the diffusion of the medium into the ALG core. The correlation between ALG concentration and the payload release is inversely proportional. The uncoated and CHIT-coated particles showed a similar release tendency, regardless of size. The most significant difference was noticed for GEL-coated particles. Interestingly, the introduction of a gelatin layer on the microparticles slightly limited the release of CUR in intestinal conditions, while in the case of macroparticles, it did not exert any substantial effect. The burst phenomena in SIF were observed only in the case of CHIT-coated microparticles, regardless of the ALG concentrations. It is clear that CUR was released from the CHIT-coated particles at a relatively fast rate, as chitosan can dissolve in SGF, whereas the rest of it, penetrating the ALG core, undergoes degradation in SIF. Finally, only the uncoated particles and GEL-coated microparticles demonstrated a significant transfer of CUR (at least 65%) to SCF. When the particles were introduced into a medium with a lower pH than the previous one, accompanied by a change of ionic strength, the interaction between the alginate chains changed slightly. The matrices were already swollen due to the repulsive interactions that had occurred in the previous medium, and as such, a burst release was observed (except for CA20UM and CA20GM). The GEL-coated macroparticles, all CHIT-coated particles, CA10UM and CA15UM liberated all payload residues during the first 2 h of the SCF experiment. The release time for the rest of the particles was up to 24 h in total. Interestingly, CA20UM, CA20GM, and CA20UX in SCF showed a similar release curve with a consistently increasing trend. Moreover, these particles released less than 60% of the encapsulated CUR within 6 h of a three-step experiment. The observed behavior of the microparticles is surprising as they contained much less payload than the macroparticles. CHIT-coated particles would be useful for carrying CUR to the intestine, whereas uncoated and GEL-coated particles for carrying it to the colon. Overall, this study reveals that the particle structural composition played an important role in controlling the CUR release profile.

Understanding the changes and factors influencing the release capacity of CUR-loaded particles is decisive in predicting and controlling the performance in various conditions. Therefore, the experimental release data were fitted and compared with two theoretical models—a hybrid model and a Korsmeyer–Peppas one. The best-fitted results are presented in Table 2 and Figure 5 (Tables S1–S3 show all fit results). It was found that for the best fit in all releasing media, R^2^_adj_ of both models were in the 0.882–0.999 range, which confirmed that the selected model fitted well with the experimental data. The H model described the CUR release profiles under simulated gastric, intestinal, and colonic conditions of GEL-coated macroparticles, regardless of the ALG concentration. H also described the release of CUR from uncoated micro- and macro-particles and CA20CX in SGF and SIF. The KP model was suitable for describing the CUR release profiles in SGF and SIF from CHIT-coated microparticles. The release profile of GEL-coated macroparticles in SGF was fitted to H, while the profile in SIF to KP. An inverse relationship was noted for CA10CX and CA15CX. In addition, KP was fitted to the CUR release profiles from each particle type, except for CHIT-coated microparticles.

Furthermore, the application of the selected models to the experimental data of CUR release from the particles makes it possible to determine the main release mechanism. According to the assumptions of Korsmeyer–Peppas for spherical particles, the value of the n coefficient (diffusion index) may suggest the following release mechanism: for n < 0.43, the release is driven by diffusion and erosion; for n = 0.43, the release mechanism is Fickian diffusion; for 0.43 < n < 0.85, the release refers to anomalous transport; and for n > 0.85, the release kinetics follow the super-case II transport [30]. In contrast, the hybrid model is more detailed and has proven effective in our earlier work on the description of the release profiles of alginate-based particles and capsules as it links the first-order kinetics of the burst phase with the Korsmeyer–Peppas model [17]. Both models selected helped to propose the mechanism of releasing payload from the particles based on the value of the n coefficient. Under simulated gastric conditions, the release mechanism was driven by the super-case II transport, regardless of the particle structure. Super-case II transport was also the predominant release mechanism under simulated intestinal conditions. The exception was CHIT-coated microparticles for which a combination of diffusion and erosion was identified as the release mechanism. The release of CUR in SCF for the vast majority of the particles was also identified as a reciprocal effect of diffusion and erosion. During the threefold change in experimental conditions, the particle matrices were subjected to intense electrostatic interactions, which caused them to swell or even partially disrupt. Thanks to this, the medium effectively penetrated the particles. Only in the case of GEL-coated microparticles was the release mechanism in SCF identified as anomalous (it involves a combination of diffusion, swelling, and degradation of the polymeric matrix)—gelatin had the greatest effect on the release mechanism.

### 2.3. In vitro Cytotoxicity of the CUR-Loaded Particles to Human Colon Adenocarcinoma Cells

**Figure 6:**
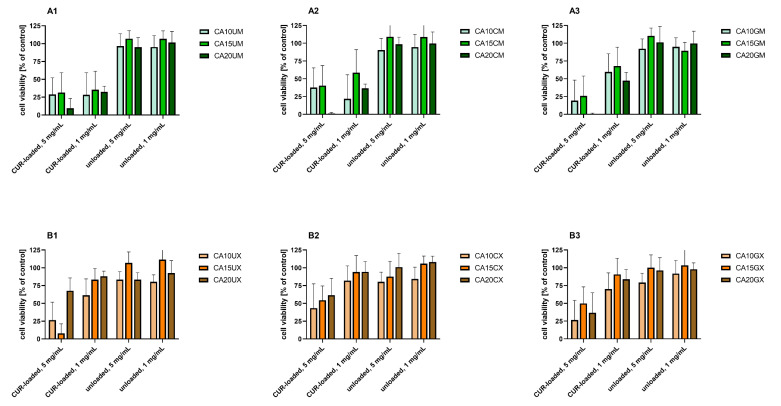
*In vitro* cytotoxicity of (**A1**) uncoated microparticles, (**A2**) CHIT-coated microparticles, (**A3**) GEL-coated microparticles, (**B1**) uncoated macroparticles, (**B2**) CHIT-coated macroparticles, (**B3**) GEL-coated macroparticles (see Table 1 for details of the particle composition). CUR-loaded and unloaded particles were added to human colon adenocarcinoma (LoVo) cells at two concentrations (1 and 5 mg/mL) for 24 h. Cell viability was evaluated using an MTT assay. The results are presented as the means ± S.D.

**Figure 7:**
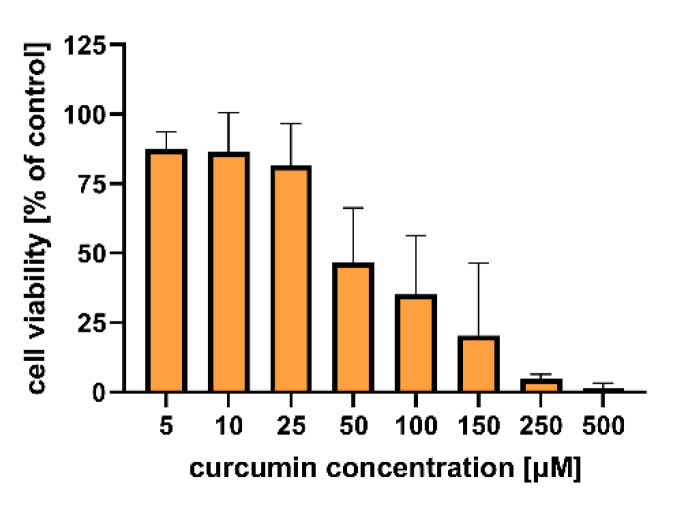
*In vitro* cytotoxicity of curcumin to human colon adenocarcinoma (LoVo) cells after 24 h of incubation. Cell viability was evaluated by an MTT assay. The results are presented as the means ± S.D.

Figure 6 shows the results of the *in vitro* study that aimed to evaluate the cytotoxicity of the particles to colon carcinoma (LoVo) cells. The cytotoxic effect of curcumin alone was assessed for comparison (Figure 7). Table S4 includes results of the statistical analysis of the significance of differences between various experimental groups. The results obtained indicated that within the studied range of concentrations, curcumin concentration of 50 µM and more significantly influenced human colon cancer cells (viability was reduced below 50% compared to the untreated control).

For all experimental setups, the empty particles were not cytotoxic to LoVo cells, as the cell viability was still above 80% compared to the untreated control. These results confirm the safety of the proposed vehicles themselves, suggesting that a rational design of the carriers—based on such natural compounds as chitosan, gelatin, and sodium alginate—enabled the fabrication of biosafe drug delivery systems. A similar effect was demonstrated by Martins et al., who designed polyelectrolyte complex beads based on N,N,N-trimethyl chitosan (TMC) and sodium alginate (ALG). The biocompatibility of such a system was evaluated in Caco-2 colon cancer cells and healthy VERO cells. For both cell lines, no significant cytotoxic effect was observed [31]. Moreover, pH-responsive k-Carrageenan/tramadol loaded UiO-66 bio-nanocomposite hydrogel beads proposed by Javanbakht et al. did not reduce the viability of human colon cells [32]. Another safe and effective carrier was described by Nia et al. The fabricated pH-sensitive carboxymethylcellulose-based bio-nanocomposite hydrogel beads, designed as a controlled amoxicillin nanocarrier for colonic bacterial infection treatment, did not reveal cytotoxicity to human umbilical vein endothelial cells (HUVECs) [33]. On the other hand, in our study, the particles loaded with curcumin induced a cytotoxic effect in human colon cancer cells. This effect was particularly strong when a higher particle concentration (5 mg/mL) was delivered. Considering the various types of vehicles, no statistically significant differences between the particles with various ALG concentrations used as a core polymer (1.0%, 1.5%, and 2.0%) were observed (*p* = 0.1901) (Table S4). However, the influence of particle size on the cytotoxic effect on LoVo cells was statistically significant (*p* < 0.0001). Cell viability was significantly lower after incubation with microparticles compared to macroparticles. This dependency was particularly visible for uncoated particles (Figure 6, B1 vs. A1), which could be explained taking into account the different release profiles of uncoated macroparticles and microparticles (Figure 5). The curcumin release was prolonged in the case of microparticles, whereas in the case of macroparticles, a burst release occurred. As prolonged drug release manner could improve a drug stability and overall therapeutic efficacy, as well as prevent particles aggregation, DDS providing sustained release profile are usually preferred [34]. In Ham’s F-12 medium, microparticles swelled to a lesser extent than the macroparticles; thus, microparticle structural changes—including the loosening of polymer chains or increasing pore diameter—were less pronounced under these conditions. Hence, the observed cytotoxicity differences could be explained in conjunction with the results of release kinetics and swelling behavior. Additionally, while analyzing various types of macroparticle coating (uncoated, chitosan, and gelatin), it was observed that cytotoxicity differences were statistically significant (*p* = 0.0044). The results revealed that chitosan-coated macroparticles were significantly less cytotoxic than both the uncoated (*p* = 0.0047) and gelatin-coated macroparticles (*p* = 0.0375). A comparison of microparticles did not show any statistically significant differences between uncoated, GEL-coated, and CHIT-coated particles (*p* = 0.1046).

The proposed systems designed for effective delivery of curcumin to the colon undoubtedly have potential demonstrated both in release kinetic studies and cytotoxicity studies. The next step is to evaluate the most promising carriers in terms of the biological effects exerted. Such an evaluation should be performed to explain in detail the mechanisms of the anticancer activity of the system. Curcumin is known for its ability to interfere with different cellular pathways and modify the production of various types of cytokines, enzymes, or growth factors [5]. Slika et al. designed nanocapsules with curcumin and demonstrated their effectiveness against human colon cancer cells both *in vitro* and *in vivo* [35]. The molecular mechanisms of action of the currently proposed CUR-loaded micro- and macro-carriers are still unrevealed and require thorough investigation.

## 3. Materials and Methods

### 3.1. Materials

Alginic acid sodium salt (medium viscosity) (ALG), gelatin from porcine skin (type A) (GEL), 0.025% trypsin, and a 0.02% EDTA solution, pepsin from porcine gastric mucosa, bile salts, SPAN80, DMSO, Dulbecco’s phosphate-buffered saline (DPBS), penicillin/streptomycin and an MTT reagent (3-(4,5-dimethyl-2-thiazolyl)-2,5-diphenyl-2H-tetrazolium bromide) were purchased from Sigma-Aldrich (Poznań, Poland). Chitosan (Mw=100–300 kDa) (CHIT) was sourced from Acros Organics (Geel, Belgium). Curcumin (for synthesis) (CUR) was received from Merck (Darmstadt, Germany). Inorganic salts, acids and hydroxides, and glacial acetic acid were of analytical grade and were sourced from Avantor (Gliwice, Poland). Ham’s F-12 medium was prepared by the Institute of Immunology and Experimental Therapy, Polish Academy of Sciences (Wrocław, Poland). Foetal bovine serum (FBS) was obtained from Lonza BioWhittaker (Basel, Switzerland). GlutaMax was purchased from Gibco, Thermo Fisher Scientific (Waltham, MA, USA). Plastic flasks, 75 cm^2^ and 96-well tissue culture plates were sourced from Nunc (Roskilde, Denmark).

### 3.2. Fabrication of the CUR-Loaded Particles

CUR was finely dispersed (homogenizer X120 (CAT, Poland)) in an ALG solution with proper concentration (1.0%, 1.5%, or 2.0%) (1:2 *w*/*w*). To prepare uncoated macroparticles, the prepared CUR–ALG mixtures were extruded by a syringe pump (Ascor Med, Poland) (50 mL/min; 22 gauge needles) into a 0.2 M CaCl_2_ solution (1:10, *v/v*). The resulting spheres were cured (at room temperature (RT), 90 min, 50 RPM), filtered, thoroughly rinsed with distilled water, and finally dried (4°C, 48 h). To prepare uncoated microparticles, the previously prepared CUR–ALG mixtures were emulsified with a 1% SPAN80 hexane solution (1:4, *v/v*) (RT, 10 min, 750 RPM). Then, the emulsion containing 0.8M CaCl_2_ and 1% SPAN80 hexane solution (1:4, *v/v*) was added dropwise. The formed microparticles were cured (RT, 60 min, 750 RPM), recovered from the emulsion, thoroughly washed, and finally dried (4 °C, 48 h). To prepare the CHIT-coated micro- and macro-particles, the uncoated particles were immersed in a 0.4% chitosan solution (dissolved in 2% acetic acid aqueous solution (*v/v*)) (RT, 15 min, 50 RPM), which was followed by their thorough rinsing with distilled water to remove an unadsorbed polysaccharide, and finally dried (4 °C, 48 h) [36]. To prepare GEL-coated micro- and macro-particles, the uncoated particles were immersed in 4.5% gelatin solution (15 min, 45°C, 50 RPM), followed by thorough particle rinsing with distilled water to remove an unadsorbed protein, as well as curing of the gelatin shell (4°C, 60 min, 50 RPM). The GEL-coated particles were filtered and finally dried (4°C, 48 h) [36].

### 3.3. Characterization of the CUR-Loaded Particles

#### 3.3.1. SEM Analysis of the Particles Surface Morphology

The surface morphology of the CUR-loaded particles was examined using scanning electron microscopy (SEM) (JSM-6601LV, JEOL, Japan) (acceleration voltage = 12 kV, 60–90× magnification for macroparticles, 120–160× magnification for microparticles).

#### 3.3.2. Calculation of the Average Size of the Particles

The average size (D) of the CUR-loaded particles of each type was calculated from the size of 100 randomly selected particles. The measurement was performed manually on all types of particles in triplicate using a polarizing microscope (41-CX, Olympus, Japan), a capture device (500MI, Ataray Ltd., Turkey) and calibrated software (Quick Photo 2.2, Promicra, Czech Republic). This number of particles was chosen as a representative sample. The size distribution graphs were prepared using the OriginPro 9.0 software (OriginLab Corporation, USA).

#### 3.3.3. Calculation of the Encapsulation Efficiency, the Moisture Content and the Swelling Index

To estimate the encapsulation efficiency (EE), hydrated CUR-loaded microparticles were fragmented and CUR was extracted with methanol (1:500, *w/v*) (RT, 48 h, 500 RPM) as the acceptor medium. EE was calculated according to the following equation:

$$\mathrm{EE}\;\left(\%\right)=\frac{m_{E}}{m_{I}}\times 100\%$$

where m_I_ is the initial mass of CUR used during the particle fabrication process, and m_E_ is the mass of the CUR encapsulated in the particles. The m_E_ for each particle type was estimated with UV-Vis measurements based on the appropriate calibration curve (λ = 420 nm) (U-2900, Hitachi, Japan), where CUR was used as a reference substance. To determine the moisture content (MC), the particles were subjected to initial drying (4 ºC, 48 h), followed by freeze-drying (–50 ºC, 96 h, 0.400 mBar), and finally kept in a vacuumed desiccator until their mass was stable. The MC was calculated using the following equation:

$$\mathrm{MC}\;\left(\%\right)=\frac{m_{\mathrm{ID}}-m_{\mathrm{FD}}}{m_{\mathrm{FD}}}\times 100\%$$

where m_ID_ is the mass of the particles post initial drying, and m_FD_ is the final mass of the particles after the full drying procedure. The swelling index (SI) of the particles was studied in Ham’s F-12 medium (1:50, *w/v*). The hydrated particles were submerged in the medium and—at predetermined time points—filtered off, centrifuged to remove any excess medium (60 s, 5000 RPM), and then weighted (ABJ 120-4NM, KERN, German). The SI was calculated using the following equation:

$$\mathrm{SI}=\frac{m_{S}-m_{\mathrm{IS}}}{m_{\mathrm{IS}}}$$

where m_IS_ is the initial mass of the particles and m_S_ is the mass of swollen particles at the selected time point.

### 3.4. FTIR Analysis

CUR, ALG, CHIT, and GEL were studied in the form they were purchased in, while the particles were first carefully minced. 20 mg of the selected substance was applied to an attenuated total reflectance (ATR) accessory equipped with a diamond crystal (IRSpirit, Shimadzu Corp., Japan) and Fourier transform infrared (FTIR) spectra were recorded (4000–400 cm^−1^, 64 scans, resolution 4 cm^−1^, RT).

### 3.5. Studies on CUR Release Profile In vitro

The particles were sequentially submerged in the following media (37 °C, 100 RPM) (1:100, *w/v*): simulated gastric fluid (SGF) (0.9% NaCl, 0.3% pepsin; pH = 2.0; 2 h), simulated intestinal fluid (SIF) (0.6% NaCl, 0.8% KCl, 0.2% CaCl_2_, 0.1% NaHCO_3_, 0.3% bile salts; pH = 7.5; 2 h) [36,37], simulated colon fluid (SCF) (0.80% NaCl, 0.02% KCl, 0.02% KH_2_PO_4_, 0.14% NaHPO_4_; pH = 6.8; 20 h) [38]. At the predetermined time points, the samples were withdrawn (1:50, *v/v*) and they were replaced with the same volume of fresh medium. After the SGF and SIF experimental stages, the particles were filtered from the medium, carefully washed with distilled water, and transferred into the subsequent medium. The withdrawn samples were mixed with methanol (1:10), centrifuged (60 s, 5000 RPM), and the amount of CUR released at a particular time point was estimated with UV-Vis measurements (λ = 420 nm) (U-2900, Hitachi, Japan). The cumulative release of CUR was calculated using the following equation:

$$\mathrm{CR}\;\left(\%\right)=\frac{m_{R}}{m_{T}}\times 100\%$$

where m_R_ is the mass of CUR released from the particles and m_T_ is the total mass of CUR liberated after 10 h of the experiment. Unloaded microparticles were used as the reference. The hybrid (H) [17] and Korsmeyer–Peppas (KP) [30] models were fitted to the experimental data using the OriginPro 9.0 software (OriginLab Corporation, USA). Below are the model equations:
$\mathrm{for}\;\mathrm{the}\;\mathrm{SIF}\;\mathrm{phase}:\;\frac{M_{t}}{M_{\infty }}=\frac{M_{H1}}{M_{\infty }}\;\left(1-e^{\left(-k_{H1}t\right)}\right)+k_{H2}t^{n_{H1}}$
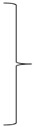
Hybrid$\mathrm{for}\;\mathrm{the}\;\mathrm{SGF}\;\mathrm{phase}:\;\frac{M_{t}}{M_{\infty }}=\frac{M_{SGF}}{M_{\infty }}+\frac{M_{H2}}{M_{\infty }}\;\left(1-e^{\left(-k_{H3}t\right)}\right)+k_{H4}t^{n_{H2}}$$\mathrm{for}\;\mathrm{the}\;\mathrm{SCF}\;\mathrm{phase}:\;\frac{M_{t}}{M_{\infty }}=\frac{M_{SIF}}{M_{\infty }}+\frac{M_{H3}}{M_{\infty }}\;\left(1-e^{\left(-k_{H5}t\right)}\right)+k_{H6}t^{n_{H3}}$
$\mathrm{SGF}\;\mathrm{phase}:\;\frac{M_{t}}{M_{\infty }}=k_{KP1}\times t^{n_{KP1}}$
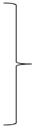
Korsmeyer–Peppas$\mathrm{SIF}\;\mathrm{phase}:\;\frac{M_{t}}{M_{\infty }}=\frac{M_{SGF}}{M{\;}_{\infty }}+k_{KP2}\times t^{n_{KP2}}$$\mathrm{SCF}\;\mathrm{phase}:\;\frac{M_{t}}{M_{\infty }}=\frac{M_{SIF}}{M{\;}_{\infty }}+k_{KP3}\times t^{n_{KP3}}$
where: M_H1_, M_H2_, and M_H3_ indicate the amounts of CUR released during the burst phase in SGF, SIF, and SCF phases, respectively; k_H1_, k_H3_ and k_H5_ are first-order rate constants for SGF, SIF and SCF phases respectively; k_H2_, k_H4_, k_H6_, k_KP1_, k_KP2_, and k_KP3_ are the Korsmeyer–Peppas release constant for SGF, SIF and SCF phases respectively; and n_H1_, n_H2_, n_H3_, n_KP1_, n_KP2_, and n_KP3_ are the release exponents for SGF, SIF and SCF phases respectively.

### 3.6. In Vitro Studies on the Cytotoxicity of CUR-Loaded Particles on Human Colon Adenocarcinoma Cells

The *in vitro* study was performed using a human colon adenocarcinoma cell line (LoVo: ATCC CCL-229) kindly gifted by the Institute of Immunology and Experimental Therapy in Wrocław (Poland). The cells were grown in Ham’s F-12 medium supplemented with 10% foetal bovine serum, 0.01% GlutaMax, and 1% antibiotics: penicillin/streptomycin. Cell cultures were grown as a monolayer on a 75 cm^2^ plastic flask and maintained in a humidified atmosphere at 37 °C and 5% CO_2_.

The cytotoxicity of the prepared particles on LoVo cells was assessed by an MTT assay. For this purpose, DMSO was used to achieve the extracts of the particles that were subsequently diluted with Ham’s F-12 medium to obtain the required concentrations used for the tests: 1.0, 2.5 and 5.0 mg/mL. The cytotoxicity of free curcumin was investigated at the following concentrations: 5, 10, 25, 50, 100, 150, 250, and 500 µM. DMSO concentration in the final solutions did not exceed 0.5% vol.; it was also validated for each experiment that it did not affect the cell viability. Fresh dilutions of the particles were prepared before each experiment.

To evaluate the particles’ cytotoxicity, cells were rinsed with DPBS, detached by trypsinization (0.025% trypsin and 0.02% EDTA) and seeded into 96-well tissue culture plates at a density of 5 × 10^4^ cells/well. After 24 h, the culture medium was replaced with the proper dilution of the CUR-loaded or empty particles and the cells were exposed to them for 24 h. Afterwards, cell viability was determined via MTT assays used to evaluate particle cytotoxicity based on differences in the cells’ mitochondrial dehydrogenase activity. For this purpose, cells were incubated for 90 min with 100 μL of the MTT reagent (3-(4,5-dimethyl-2-thiazolyl)-2,5-diphenyl-2*H*-tetrazolium bromide) at 37 °C. Formazan crystals were then dissolved using 100 μL of acidic isopropanol and by mixing. The absorbance was measured at 570 nm using a multiwell plate reader (GloMax^®^ Discover Microplate Reader, Promega, USA). The results were expressed as the percentage of the viability of the treated cells related to untreated control cells with normal mitochondrial activity, considered as 100%.

### 3.7. Statistical Analysis

Statistical analysis of the obtained results was performed using the Origin Pro 9.0 software (OriginLab Corporation, Northampton, MA, USA), as well as the GraphPad Prism 9.1.2. software (GraphPad Software, San Diego, CA, USA). All experiments were carried out at least in triplicate (3 independent repetitions) and at least three samples were measured per experiment (n = 9). The results are expressed as a mean ± S.D. A one-way ANOVA with a post hoc Tukey HSD test was applied and the differences among groups were considered statistically significant with *p* < 0.05.

## 4. Conclusions

The study has revealed differences between various types of vehicles designed to deliver curcumin to the colon. The following conclusions can be drawn based on the results obtained:

- The encapsulation efficiency differed significantly between micro- and macro-particles. Regardless of the structural composition, the microparticles encapsulated much less payload than the macroparticles.
- The structure of the particle (concentration of the core, polymer, presence and type of the external shell) affects its performance in simulated gastrointestinal conditions.
- Release profile studies revealed that CHIT-coated particles would be useful for carrying CUR to the intestine, as chitosan can dissolve in SGF and the burst phenomena were observed in SIF. On the other hand, uncoated and GEL-coated particles demonstrated a significant transfer of CUR to SCF and may be considered as useful systems for carrying CUR to the colon.
- The unloaded carriers were not cytotoxic to human colon adenocarcinoma cells, while the curcumin-loaded vehicles impaired their viability.
- The viability of colon cancer cells was more significantly reduced after incubation with curcumin-loaded microparticles than macroparticles.
- The potential anticancer activity of the proposed curcumin-loaded carriers requires further investigation.

The ultimate conclusion is that we can select gelatin-coated or uncoated microparticles as the most promising carriers for the delivery of curcumin to the colon. In the future, more detailed biological studies will be performed to evaluate the anticancer potential of such systems and thoroughly explain their mechanism of action.

## Figures and Tables

**Table 2:** Kinetic parameters of hybrid (H) and Korsmeyer–Peppas (KP) models estimated from the fitting to experimental data received during the simulated gastric fluid (SGF), simulated intestine fluid (SIF), and simulated colon fluid (SCF) stages of CUR *in vitro* release. Bold type indicates the best fit. For details of the particle composition, see Table 1. For a detailed description of the kinetic parameters, see Section 3.5.

	H-Model					KP-Model		
Particle	SGF Stage							
	M_H1_	k_H1_	k_H2_	n_H1_	R_H1_^2^	k_KP1_	n_KP1_	R_KP1_^2^
CA10UM	3.01	3.4 × 10^−4^	3.6 × 10^−6^	2.90	0.952			
CA15UM	0.22	1.3 × 10^−2^	2.6 × 10^−1^	0.86	0.996			
CA20UM	0.00	5.0 × 10^−2^	5.3 × 10^−7^	3.87	0.961			
CA10CM						7.4 × 10^−2^	1.14	0.910
CA15CM						9.4 × 10^−2^	1.02	0.988
CA20CM						8.6 × 10^−2^	1.06	0.979
CA10GM	0.03	6.1 × 10^−1^	9.8 × 10^−2^	1.04	0.968			
CA15GM	0.01	1.3 × 10^−7^	1.2 × 10^−14^	6.69	0.901			
CA20GM	0.01	1.2 × 10^−1^	2.0 × 10^−16^	7.02	0.981			
CA10UX	0.07	1.0 × 10^−3^	1.7 × 10^−4^	0.87	0.979			
CA15UX	0.01	1.7 × 10^−3^	1.5 × 10^−8^	3.43	0.978			
CA20UX	0.01	4.4 × 10^2^	6.1 × 10^−4^	1.84	0.969			
CA10CX						2.9 × 10^−4^	2.17	0.917
CA15CX						1.2 × 10^−1^	0.86	0.926
CA20CX	0.00	5.4 × 10^−2^	5.1 × 10^−18^	7.57	0.991			
CA10GX	0.00	2.0 × 10^10^	3.8 × 10^−4^	2.72	0.956			
CA15GX	0.00	5.0 × 10^−2^	3.8 × 10^−5^	2.12	0.962			
CA20GX	0.01	1.0 × 10^−3^	1.0 × 10^−3^	0.89	0.854			
**Particle**	**SIF Stage**							
	**M_H2_**	**k_H3_**	**k_H4_**	**n_H2_**	**R_H2_^2^**	**k_KP2_**	**n_KP2_**	**R_KP2_^2^**
CA10UM	0.005	1.0 × 10^−3^	7.5 × 10^−4^	1.30	0.989			
CA15UM	0.005	1.0 × 10^−3^	3.8 × 10^−3^	0.87	0.975			
CA20UM	0.005	1.0 × 10^−3^	1.8 × 10^−3^	1.04	0.950			
CA10CM						2.8 × 10^−1^	0.27	0.891
CA15CM						2.1 × 10^−1^	0.29	0.908
CA20CM						3.2 × 10^−2^	0.42	0.984
CA10GM	0.005	1.0 × 10^−3^	1.0 × 10^−5^	2.16	0.965			
CA15GM	0.005	1.0 × 10^−3^	2.9 × 10^−4^	1.46	0.993			
CA20GM	0.005	1.0 × 10^−3^	2.9 × 10^−3^	0.86	0.996			
CA10UX	0.005	1.0 × 10^−3^	5.6 × 10^−4^	1.38	0.976			
CA15UX	0.005	1.0 × 10^−3^	1.0 × 10^−5^	2.14	0.967			
CA20UX	0.005	1.0 × 10^−3^	2.3 × 10^−5^	1.72	0.988			
CA10CX	0.005	1.0 × 10^−3^	4.3 × 10^−7^	1.10	0.882			
CA15CX	0.005	1.0 × 10^−3^	3.6 × 10^−5^	2.12	0.894			
CA20CX	0.005	1.0 × 10^−3^	1.0 × 10^−5^	2.32	0.955			
CA10GX						1.8 × 10^−7^	3.25	0.996
CA15GX	0.005	1.0 × 10^−3^	1.7 × 10^−4^	1.70	0.928			
CA20GX						1.2 × 10^−8^	3.53	0.944
**Particle**	**SCF Stage**							
	**M_H3_**	**k_H5_**	**k_H6_**	**n_H3_**	**R_H3_^2^**	**k_KP3_**	**n_KP3_**	**R_KP3_^2^**
CA10UM						4.7 × 10^−1^	0.14	0.997
CA15UM						3.1 × 10^−1^	0.21	0.997
CA20UM						2.9 × 10^−1^	0.08	0.972
CA10CM						8.8 × 10^−1^	0.02	0.999
CA15CM						7.6 × 10^−1^	0.04	0.993
CA20CM						7.3 × 10^−1^	0.02	0.999
CA10GM	0.005	1.0 × 10^−3^	2.6 × 10^−4^	1.41	0.971			
CA15GM	0.005	1.0 × 10^−3^	4.2 × 10^−2^	0.86	0.991			
CA20GM	0.005	1.0 × 10^−3^	3.1 × 10^−3^	1.04	0.943			
CA10UX						4.0 × 10^−1^	0.14	0.997
CA15UX						2.9 × 10^−1^	0.14	0.983
CA20UX						1.1 × 10^−1^	0.29	0.968
CA10CX						8.2 × 10^−1^	0.04	0.999
CA15CX						8.5 × 10^−1^	0.02	0.999
CA20CX						7.0 × 10^−1^	0.03	0.994
CA10GX	na	na	na	na	na	na	na	na
CA15GX						5.3 × 10^−1^	0.12	0.995
CA20GX						4.4 × 10^−1^	0.18	0.966

## Data Availability

All data presented in this study are included in the published article or are available on request from the corresponding author.

## Supplementary Materials

The following are available online, S1.2 (Methods): UV-Vis analysis; Figure S1: UV-Vis spectra recorded for CUR dissolved in acetone (C = 10 μg/mL) and extracted from CA10UM and CA10UX particles; Table S1: Kinetic parameters of hybrid (H) and Korsmeyer–Peppas (KP) models estimated from the fitting to experimental data received during the simulated gastric fluid (SGF) stage of the *in vitro* release of CUR. Bold type indicates the best fit; Table S2: Kinetic parameters of hybrid (H) and Korsmeyer–Peppas (KP) models estimated from the fitting to experimental data received during the simulated intestinal fluid (SIF) stage of the *in vitro* release of CUR. Bold type indicates the best fit; Table S3: Kinetic parameters of hybrid (H) and Korsmeyer–Peppas (KP) models estimated from the fitting to experimental data received during the simulated colon fluid (SCF) stage of the *in vitro* release of CUR. Bold type indicates the best fit; Table S4: One-way ANOVA multiple comparison results; red color denotes statistically significant differences.

## Sample Availability

Samples of microparticles and macroparticles are available from the authors upon reasonable request.
