# Supplementary material for: Microparticles vs. Macroparticles as Curcumin Delivery Vehicles: Structural Studies and Cytotoxic Effect in Human Adenocarcinoma Cell Line (LoVo)
Source: Molecules. 2021 Oct 6;26(19):6056. doi: 10.3390/molecules26196056 (PMC8512134; doi:10.3390/molecules26196056)
Supplement: Supplementary file 1 [file molecules-26-06056-s001.zip › molecules-1380337-supplementary.pdf]

S1. Methods

S1.2 UV-Vis analysis

CUR was extracted from wet CA10UM and CA10UX with acetone (1:500, w/v) (48 h, 500 RPM, room temperature). Then, the solutions were filtered, the solvent was evaporated, and CUR_{CA10UM} and CUR_{CA10UX} were again dissolved in acetone to receive a concentration of 10 µg/ml. CUR_{REF} (a reference substance) was dissolved in acetone (C = 10 µg/ml). The UV-Vis spectra were recorded (U-2900, Hitachi, Japan) (300 – 1000 nm, RT, quartz cuvette).

S2. Results

S2.1 Spectroscopic studies – UV-Vis

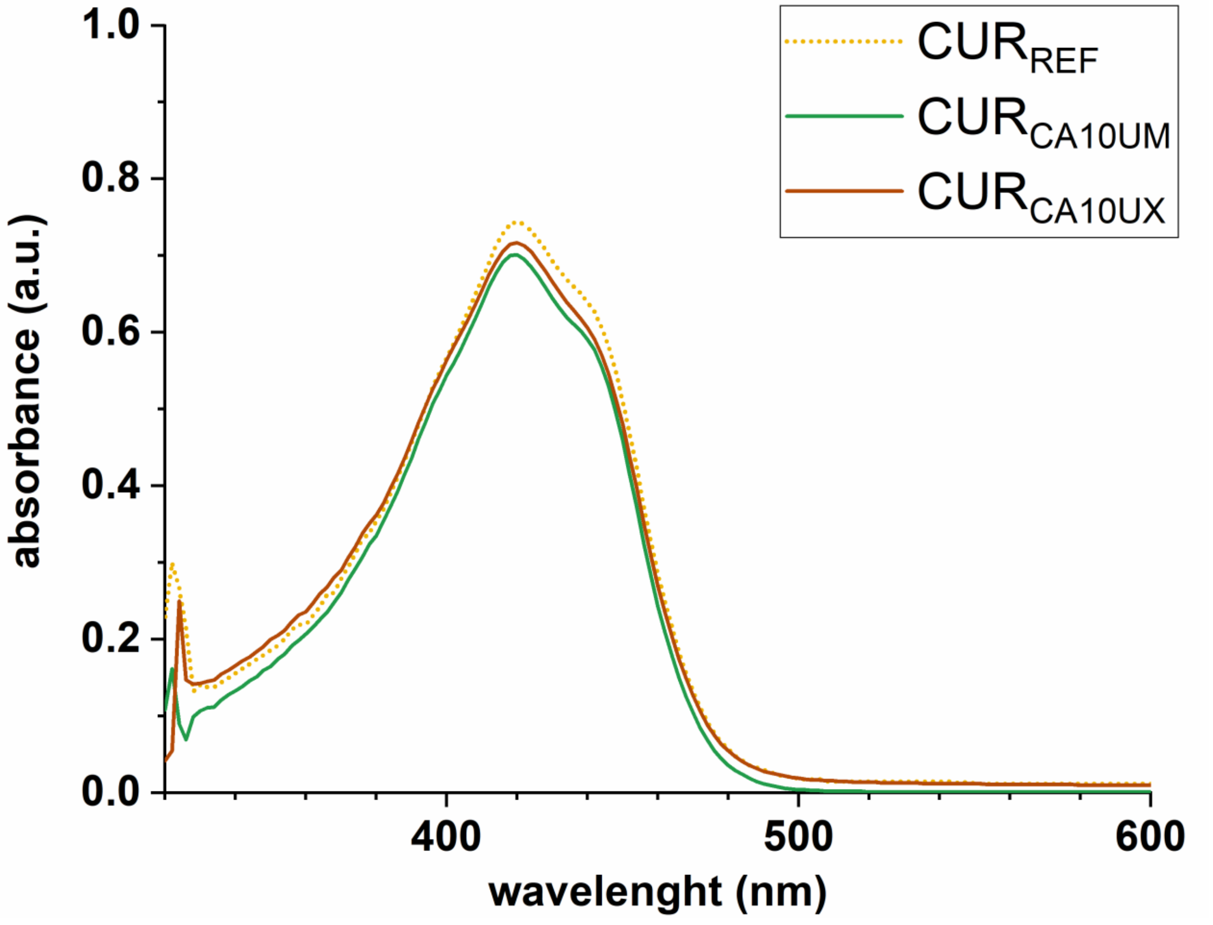

Figure S1. UV-Vis spectra recorded for CUR dissolved in acetone (C = 10 µg/ml) and extracted from CA10UM and CA10UX particles.

S2.2 Curcumin release kinetic studies

Table S1. Kinetic parameters of hybrid (H) and Korsmeyer-Peppas (KP) models estimated from the fitting to experimental data received during the simulated gastric fluid (SGF) stage of in vitro release of CUR. Bold type indicates the best fit.

particle	H-model					KP-model		
	M_{H1}	k_{H1}	k_{H2}	n_{H1}	R_{H1}^2	k_{KP1}	n_{KP1}	R_{KP1}^2
CA10UM	**3.01**	**3.4E-04**	**3.6E-06**	**2.90**	**0.952**	2.5E-01	0.73	0.944
CA15UM	**0.22**	**1.3E-02**	**2.6E-01**	**0.86**	**0.996**	5.1E-01	0.59	0.966
CA20UM	**0.00**	**5.0E-02**	**5.3E-07**	**3.87**	**0.961**	4.8E-03	1.59	0.836
CA10CM	0.06	7.0E-03	3.9E-02	1.22	0.821	**7.4E-02**	**1.14**	**0.910**
CA15CM	0.31	3.0E-03	7.9E-06	2.12	0.977	**9.4E-02**	**1.02**	**0.988**
CA20CM	0.21	1.3E-03	2.7E-02	0.97	0.749	**8.6E-02**	**1.06**	**0.979**
CA10GM	**0.03**	**6.1E+01**	**9.8E-02**	**1.04**	**0.968**	9.1E-01	0.55	0.953
CA15GM	**0.01**	**1.3E+07**	**1.2E-14**	**6.69**	**0.901**	6.2E-01	0.17	0.566
CA20GM	**0.01**	**1.2E-01**	**2.0E-16**	**7.02**	**0.981**	4.0E-01	0.21	0.910
CA10UX	**0.07**	**1.0E-03**	**1.7E-04**	**0.87**	**0.979**	3.2E-02	0.66	0.780
CA15UX	**0.01**	**1.7E-01**	**1.5E-08**	**3.43**	**0.978**	1.1E-01	0.33	0.958
CA20UX	**0.01**	**4.4E+02**	**6.1E-04**	**1.84**	**0.969**	8.4E-02	0.86	0.820
CA10CX	0.00	1.2E-08	9.9E-04	1.91	0.827	**2.9E-04**	**2.17**	**0.917**
CA15CX	0.01	1.3E-01	1.1E-02	0.97	0.857	**1.2E-01**	**0.86**	**0.926**
CA20CX	**0.00**	**5.4E-02**	**5.1E-18**	**7.57**	**0.991**	8.9E-02	0.37	0.927
CA10GX	**0.00**	**2.0E+10**	**3.8E-04**	**2.72**	**0.956**	2.0E-01	0.13	0.845
CA15GX	**0.00**	**5.0E-02**	**3.8E-05**	**2.12**	**0.962**	1.3E-03	1.23	0.937
CA20GX	**0.01**	**1.0E-03**	**1.0E-03**	**0.89**	**0.854**	1.0E-03	1.32	0.484

Table S2. Kinetic parameters of hybrid (H) and Korsmeyer-Peppas (KP) models estimated from the fitting to experimental data received during the simulated intestinal fluid (SIF) stage of in vitro release of CUR. Bold type indicates the best fit.

particle	H-model					KP-model		
	M_{H2}	k_{H3}	k_{H4}	n_{H2}	R_{H2}^2	k_{KP2}	n_{KP2}	RK_{P2}^2
CA10UM	**0.005**	**1.0E-03**	**7.5E-04**	**1.30**	**0.989**	7.6E-03	0.86	0.922
CA15UM	**0.005**	**1.0E-03**	**3.8E-03**	**0.87**	**0.975**	5.2E-02	0.34	0.903
CA20UM	**0.005**	**1.0E-03**	**1.8E-03**	**1.04**	**0.950**	2.4E-02	0.54	0.861
CA10CM	0.005	1.0E-03	1.7E-01	0.33	0.831	**2.8E-01**	**0.27**	**0.891**
CA15CM	0.005	1.0E-03	1.3E-01	0.35	0.867	**2.1E-01**	**0.29**	**0.908**
CA20CM	0.005	1.0E-03	5.0E-02	0.57	0.924	**3.2E-02**	**0.42**	**0.984**
CA10GM	**0.005**	**1.0E-03**	**1.0E-05**	**2.16**	**0.965**	8.8E-03	0.80	0.674
CA15GM	**0.005**	**1.0E-03**	**2.9E-04**	**1.46**	**0.993**	1.4E-03	1.12	0.964
CA20GM	**0.005**	**1.0E-03**	**2.9E-03**	**0.86**	**0.996**	6.3E-03	0.67	0.992
CA10UX	**0.005**	**1.0E-03**	**5.6E-04**	**1.38**	**0.976**	6.8E-04	1.34	0.974
CA15UX	**0.005**	**1.0E-03**	**1.0E-05**	**2.14**	**0.967**	1.6E-06	2.56	0.963
CA20UX	**0.005**	**1.0E-03**	**2.3E-05**	**1.72**	**0.988**	3.5E-02	0.23	0.622
CA10CX	**0.005**	**1.0E-03**	**4.3E-03**	**1.10**	**0.882**	5.0E-03	1.08	0.851
CA15CX	**0.005**	**1.0E-03**	**3.6E-05**	**2.12**	**0.894**	1.1E-06	2.85	0.854
CA20CX	**0.005**	**1.0E-03**	**1.0E-05**	**2.32**	**0.955**	2.4E-08	3.59	0.993
CA10GX	0.005	1.0E-03	1.0E-05	2.39	0.980	**1.8E-07**	**3.25**	**0.996**

CA15GX	**0.005**	**1.0E-03**	**1.7E-04**	**1.70**	**0.928**	1.4E-05	2.19	0.876
CA20GX	0.005	1.0E-03	1.0E-05	2.09	0.790	**1.2E-08**	**3.53**	**0.944**

Table S3. Kinetic parameters of hybrid (H) and Korsmeyer-Peppas (KP) models estimated from the fitting to experimental data received during the simulated colon fluid (SCF) stage of in vitro release of CUR. Bold type indicates the best fit.

**particle**	**H-model**					**KP-model**		
	M_{H3}	k_{H5}	k_{H6}	n_{H3}	R_{H3}^2	k_{KP3}	n_{KP3}	R_{KP3}^2
CA10UM	0.005	1.0E-03	3.3E-02	0.58	0.990	**4.7E-01**	**0.14**	**0.997**
CA15UM	0.005	1.0E-03	6.9E-02	0.43	0.948	**3.1E-01**	**0.21**	**0.997**
CA20UM	0.005	1.0E-03	1.7E-05	1.92	0.975	**2.9E-01**	**0.08**	**0.972**
CA10CM	0.005	1.0E-03	1.0E-02	0.46	0.957	**8.8E-01**	**0.02**	**0.999**
CA15CM	0.005	1.0E-03	2.0E-04	1.46	0.986	**7.6E-01**	**0.04**	**0.993**
CA20CM	0.005	1.0E-03	3.2E-03	0.65	0.962	**7.3E-01**	**0.02**	**0.999**
CA10GM	**0.005**	**1.0E-03**	**2.6E-04**	**1.41**	**0.971**	4.1E-01	0.08	0.662
CA15GM	**0.005**	**1.0E-03**	**4.2E-02**	**0.86**	**0.991**	3.3E-01	0.15	0.990
CA20GM	**0.005**	**1.0E-03**	**3.1E-03**	**1.04**	**0.943**	6.6E-02	0.42	0.842
CA10UX	0.005	1.0E-03	2.5E-02	0.59	0.990	**4.0E-01**	**0.14**	**0.997**
CA15UX	0.005	1.0E-03	8.7E-03	0.77	0.966	**2.9E-01**	**0.14**	**0.983**
CA20UX	0.005	1.0E-03	1.5E-02	0.65	0.907	**1.1E-01**	**0.29**	**0.968**
CA10CX	0.005	1.0E-03	2.0E-02	0.46	0.920	**8.2E-01**	**0.04**	**0.999**
CA15CX	0.005	1.0E-03	3.3E-03	0.75	0.988	**8.5E-01**	**0.02**	**0.999**
CA20CX	0.005	1.0E-03	1.0E-05	2.01	0.984	**7.0E-01**	**0.03**	**0.994**
CA10GX	na	na	na	na	na	na	na	na
CA15GX	0.005	1.0E-03	7.6E-02	0.37	0.874	**5.3E-01**	**0.12**	**0.995**
CA20GX	0.005	1.0E-03	1.6E-01	0.33	0.824	**4.4E-01**	**0.18**	**0.966**

Table S4. One-way ANOVA multiple comparison results; red colour denotes statistically significant differences;

a) comparisons between different concentrations of sodium alginate (2.0 % vs. 1.5 % vs. 1.0 %):

Number of families	1				
Number of comparisons per family	3				
Alpha	0,05				
Tukey's multiple comparisons test	Mean Diff,	95,00% CI of diff,	Below threshold?	Summary	Adjusted P Value
20 vs. 15	3,674	-7,624 to 14,97	No	ns	0,7245
20 vs. 10	8,335	-2,808 to 19,48	No	ns	0,1845
15 vs. 10	4,661	-4,785 to 14,11	No	ns	0,4772

b) comparisons between different types of particles (macro- and microparticles uncoated, coated with chitosan or coated with gelatin;

UM vs. UX vs. CM vs. CX vs. GM vs. GX):

Number of families	1					
Number of comparisons per family	15					
Alpha	0,05					
Tukey's multiple comparisons test	Mean Diff,	95,00% CI of diff,	Below threshold?	Summary	Adjusted P Value	
UM vs. UX	-7,402	-23,96 to 9,158	No	ns	0,7954	A-B
UM vs. CM	-7,043	-24,80 to 10,71	No	ns	0,8657	A-C
UM vs. CX	-25,12	-41,98 to -8,270	Yes	***	0,0004	A-D
UM vs. GM	6,855	-10,99 to 24,70	No	ns	0,8809	A-E
UM vs. GX	-11,11	-27,96 to 5,743	No	ns	0,4107	A-F
UX vs. CM	0,3585	-16,71 to 17,43	No	ns	>0,9999	B-C
UX vs. CX	-17,72	-33,85 to -1,588	Yes	*	0,0219	B-D
UX vs. GM	14,26	-2,910 to 31,42	No	ns	0,1662	B-E
UX vs. GX	-3,708	-19,84 to 12,42	No	ns	0,9862	B-F
CM vs. CX	-18,08	-35,43 to -0,7241	Yes	*	0,0356	C-D
CM vs. GM	13,90	-4,422 to 32,22	No	ns	0,2527	C-E
CM vs. GX	-4,066	-21,42 to 13,29	No	ns	0,9850	C-F
CX vs. GM	31,98	14,53 to 49,43	Yes	****	<0,0001	D-E

CX vs. GX	14,01	-2,420 to 30,45	No	ns	0,1443	D-F
GM vs. GX	-17,96	-35,41 to -0,5151	Yes	*	0,0394	E-F

c) comparisons between different types of macroparticles (uncoated, coated with chitosan or coated with gelatin; UX vs. CX vs. GX):

Number of families	1					
Number of comparisons per family	3					
Alpha	0,05					
Tukey's multiple comparisons test	Mean Diff,	95,00% CI of diff,	Below threshold?	Summary	Adjusted P Value	
UX vs. CX	-17,72	-30,84 to -4,598	Yes	**	0,0047	B-D
UX vs. GX	-3,708	-16,83 to 9,415	No	ns	0,7827	B-F
CX vs. GX	14,01	0,6459 to 27,38	Yes	*	0,0375	D-F

d) comparisons between different types of microparticles (uncoated, coated with chitosan or coated with gelatin; UM vs. CM vs. GM):

Number of families	1					
Number of comparisons per family	3					
Alpha	0,05					
Tukey's multiple comparisons test	Mean Diff,	95,00% CI of diff,	Below threshold?	Summary	Adjusted P Value	
UM vs. CM	-7,043	-21,93 to 7,848	No	ns	0,5038	A-C
UM vs. GM	6,855	-8,113 to 21,82	No	ns	0,5257	A-E
CM vs. GM	13,90	-1,469 to 29,26	No	ns	0,0852	C-E
